# Operation Mechanism of a MoS_2_/BP Heterojunction FET

**DOI:** 10.3390/nano8100797

**Published:** 2018-10-07

**Authors:** Sung Kwan Lim, Soo Cheol Kang, Tae Jin Yoo, Sang Kyung Lee, Hyeon Jun Hwang, Byoung Hun Lee

**Affiliations:** 1 Center for Emerging Electronic Devices and Systems (CEEDS), GIST, 123 Cheomdan-gwagiro, Buk-gu, Gwangju 61005, Korea; lsk8410@gist.ac.kr (S.K.L.); soocheol@gist.ac.kr (S.C.K.); tjyoo123@gist.ac.kr (T.J.Y.); leesk@gist.ac.kr (S.K.L.); hhjune@gist.ac.kr (H.J.H.); 2 Department of Nanobio Materials and Electronics, GIST, 123 Cheomdan-gwagiro, Buk-gu, Gwangju 61005, Korea; 3 School of Materials and Science Engineering, GIST, 123 Cheomdan-gwagiro, Buk-gu, Gwangju 61005, Korea

**Keywords:** MoS_2_, black phosphorus, 2D/2D heterojunction, junction FET, tunneling diode, tunneling FET, band-to-band tunneling (BTBT)

## Abstract

The electrical characteristics and operation mechanism of a molybdenum disulfide/black phosphorus (MoS_2_/BP) heterojunction device are investigated herein. Even though this device showed a high on-off ratio of over 1 × 10^7^, with a lower subthreshold swing of ~54 mV/dec and a 1fA level off current, its operating mechanism is closer to a junction field-effect transistor (FET) than a tunneling FET. The off-current of this device is governed by the depletion region in the BP layer, and the band-to-band tunneling current does not contribute to the rapid turn-on and extremely low off-current.

## 1. Introduction

Tunneling field-effect transistors (tFETs) have been studied as an alternative device for silicon MOSFET enabling very sharp turn-on which is required to reduce the operation voltage and the system power consumption. tFETs utilize band-to-band tunneling (BTBT) from a source to a channel, and an off-current is maintained using a P-N-N or N-P-P-type channel-doping profile [1,2,3,4,5]. When BTBT occurs in this channel-doping profile, the carriers from the source are injected directly into the channel and transported to the drain. When BTBT is not possible, the carrier cannot be injected into the drain because of the barrier formed in the channel region. In this device, the tunneling distance should be minimized to allow the tunneling current to rapidly increase. Thus, a very sharp P-N junction should be formed. The performances of experimental tunnel FETs reported in the literature have not reached their theoretical limit, primarily due to graded doping profiles and interface traps [3,6]. For an ideal BTBT current flow, an atomically sharp interface with minimal interface states is necessary. Fortunately, these requirements can be easily satisfied using transition metal dichalcogenide (TMD) materials because the various choices of band gaps and band alignment combinations make the stack of two-dimensional (2D) materials an ideal candidate for tunneling FETs [7,8,9,10,11]. Thus, a variety of stacks, including molybdenum disulfide (MoS_2_)/tungsten diselenide (WSe_2_), tin diselenide (SnSe_2_)/WSe_2_, MoS_2_/black phosphorus (BP), and SnSe_2_/BP have been investigated [12,13,14,15,16,17,18,19]. Most of these studies explain that the turn-on mechanism is due to the BTBT, and the turn off mechanism is due to the band misalignment.

In this work, we fabricated a heterojunction FET, using a multilayer MoS_2_ and a thick black-phosphorus stack with a back gate structure, and investigated the operation mechanism. This system was chosen because a MoS_2_/BP stack is suitable for broken bandgap device fabrication. Our analysis revealed that the operation mechanism of this heterojunction FET is quite different from what has been reported in the literature. The off-current is dominated by the depletion in the BP layer, and the subthreshold swing is related to the reduction of the depletion region. The BTBT current only contributes to the hump in the drain current. 

## 2. Materials and Methods

**Figure 1:**
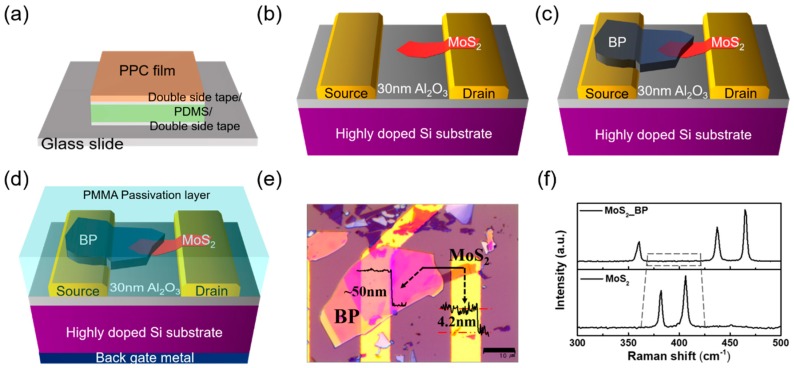
(**a**) Schematic of stamp (polypropylene carbonate (PPC)/double-sided tape/polydimethylsiloxane (PDMS)/double-sided tape/glass slide), with the 2D flake transferred directly onto the PPC film. (**b**) MoS_2_ transferred onto the drain electrode (5-nm/45-nm Ti/Au) and gate oxide (30-nm Al_2_O_3_). (**c**) Black phosphorus (BP) flake transferred quickly to the substrate using the same method. (**d**) Device passivated using polymethylmethacrylate (PMMA) film. (**e**) Optical image of MoS_2_/BP (4.2 nm/50 nm) heterojunction. (**f**) The thickness of flakes was measured using Raman spectra (using a 514-nm laser) of the molybdenum disulfide (MoS_2_)/BP stack. The lower panel shows the Raman spectra of the MoS_2_ flake (the $E_{2g}^{1}$ peak at 382.29 cm^−1^ and the A_1g_ peak at 406.25 cm^−1^).

The fabrication processes of the MoS_2_/BP heterojunction FET are shown in Figure 1. Figure 1a shows the structure of a stamp used to transfer 2D flakes. Polypropylene carbonate (PPC) (Sigma-Aldrich, CAS 25511-85-7, Sigma-Aldrich, CAS 25511-85-7, St. Louis, MO, USA) was used to pick up and transfer the flakes of MoS_2_ and BP at a low temperature [18,20,21]. Since the flakes are easily damaged during the detachment process, and some of 2D materials, e.g., SnSe_2_, hafnium diselenide (HfSe_2_), and BP, can be oxidized during the transfer or device fabrication [22,23,24], we modified the fabrication process to directly transfer the flakes to the PPC film to minimize the damage and to reduce the air exposure time. Both sides of a handmade polydimethylsiloxane (PDMS) sheet were treated with ozone plasma for 10 min to improve the adhesion of the double-sided tape to the PDMS sheet. The PPC film (15% solution in Anisole) was coated onto the stack of tape/PDMS/tape and cured on a hot plate at 100 °C for 10 min. Then, the PPC/tape/PDMS/tape sheet was placed on a glass slide patterned with align keys. Figure 1b–d show the rest of the device fabrication process. 

The source and drain electrodes (5-nm Ti/45-nm Au) were formed on a 30-nm aluminum oxide (Al_2_O_3_)/highly doped P-type silicon substrate using e-beam evaporation and photolithography. In this experiment, MoS_2_ was used as the channel material with BP as the source material. Exfoliated MoS_2_ flakes were transferred to the PPC film from a bulk crystal using commercial adhesive tape, and then transferred onto the drain electrodes using a dry transfer system at 80 °C. The selected BP flake was also transferred onto the source electrode using the same process, while carefully overlapping the BP flake onto the MoS_2_ flake that was already connected to the drain electrode. Since the BP flake could be easily oxidized in air [24], a polymethylmethacrylate (PMMA, 950 K 4 A, Microchem, Westborough, MA, USA) coating was applied, followed by thermal annealing at 180 °C for 5 min to eliminate the solvent. Figure 1e shows an optical microscope image of the device. The thickness of the MoS_2_, measured with atomic force microscopy, was 4.2 nm and the BP thickness was ~50 nm. Figure 1f shows the Raman spectrum of the BP/MoS_2_, measured from the overlapped region. The characteristic BP peaks were observed at 360.65 ($A_{g}^{1}$), 437.3 (B_2g_), and 464.4 ${(A}_{g}^{2}$) cm^−1^; however, the Raman peak of the MoS_2_ was not observed in this spectrum because the BP layer was very thick. The Raman spectrum of the MoS_2_ shown in the lower part of Figure 1f was measured from the region not overlapping the BP layer.

## 3. Results and Discussion

**Figure 2:**
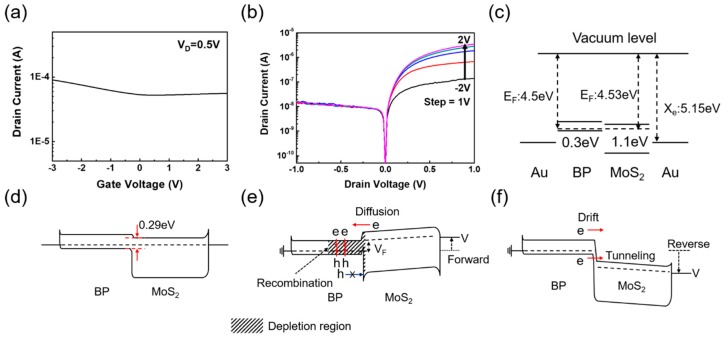
(**a**) Transfer characteristic of the thick-layer BP field-effect transistor (FET). (**b**) Electrical characteristics of a MoS_2_/BP diode following the gate voltage. Band structure of the MoS_2_/BP, (**c**) before contact, (**d**) at the equilibrium state, and (**e**) with a forward rectifying condition with negative bias applied to the MoS_2_ electrode. The holes from BP cannot overcome the high barrier at the forward bias and the electrons from MoS_2_ diffuses into BP, generating a depletion region. (**f**) Reverse bias condition with positive bias applied to the MoS_2_ electrode. The current is primarily due to the drift of minority carriers, as well as the tunneling carriers from the BP side.

First, the electrical characteristics of the MoS_2_/BP diode were measured using a parameter analyzer (Keithely 4200, Santa Rosa, CA, USA). The TMD materials show different electrical characteristics depending on the thickness of the layer. When the BP is very thick, no significant gate modulation is observed, as shown in Figure 2a. In fact, this characteristic is beneficial for device operation because the high current level with a small gate modulation means that the BP layer can be used as a good contact material with a bandgap.

Figure 2b shows the diode characteristics of the MoS_2_/BP heterojunction at different gate biases, from −2 to 2 V with a gate bias step of 1 V. While the potential of the BP layer is almost fixed to the source–drain bias, the Fermi level of the MoS_2_ layer shows a reasonable gate modulation for both single layers and multilayers [25]. As the gate bias increased from −2 V, the rectification characteristics at the MoS_2_/BP junction seemed to improve because the barrier height at the MoS_2_/BP interface increased. These characteristics can be explained more intuitively with a band diagram. The ideal band structure of a MoS_2_/BP stack before stacking is shown in Figure 2c. The work function of the 2D materials is measured differently depending on the measurement environment due to its high surface energy. We assumed that the Fermi levels of the MoS_2_ and BP are 4.53 and 4.5 eV, respectively [26,27]. After the stacking, the MoS_2_/BP heterojunction forms a staggered (type II) band alignment at an equilibrium state, with a very small barrier on the conduction band side, as shown in Figure 2d. Theoretically, the effective band gap, which is the difference between the conduction band of MoS_2_ and the valence band of BP, formed at the MoS_2_/BP junction is 0.29 eV. The effective band gap is modulated by the drain bias during the diode type operation. 

Even though the doping profile of a MoS_2_/BP junction is similar to a P-N junction, the carrier conduction mechanisms are quite different. When a negative drain bias is applied, the Fermi level of the MoS_2_ shifts upward (forward bias for a P-N junction) and the majority carriers from MoS_2_ flow into the BP layer; however, the holes in the BP layer cannot be transferred to the MoS_2_ layer, due to the high barrier height. As a result, the recombination of electrons and holes at the BP side generates a depletion region, which is balanced by the electron influx and the resistance increase, due to the depletion width increase. Hence, an almost constant current of ~10 nA is maintained in our device. In the case of a silicon P-N junction, the current increases exponentially at forward bias.

On the other hand, when the drain bias is positive (reverse bias for a P-N junction), minority carriers from the BP and MoS_2_ layers start to flow to opposite sides, driven by the electric field. Moreover, depending on the drain bias, the tunneling component may also contribute to the drain current. The current flow, shown in Figure 2b, saturates at a high drain bias because the current flow is limited by the minority carrier supply. Unlike a P-N junction, where the diffusion of the majority carriers is the primary conduction mechanism, the drift of minority carriers is the primary conduction mechanism in this bias region. Many prior studies have correctly noted this difference; however, in our opinion, they did not carefully consider the off-current mechanism [13,14,16,18,19]. Most of prior works explained that the off state is due to the band shift closing the direct tunneling window, but they did not consider that the gate bias region—causing extremely low off current—did not match the gate bias region of the direct tunneling current. 

**Figure 3:**
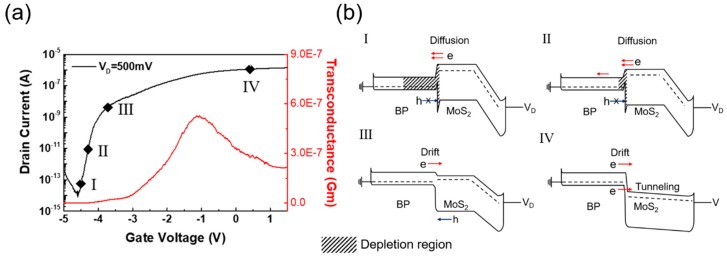
Electrical properties and current flow mechanism of a MoS_2_/BP heterojunction FET at V_D_ = 500 mV. (**a**) Transfer characteristics and transconductance (G_m_). (**b**) Band diagrams showing the states at different gate bias regions.

Figure 3a shows the transfer characteristics of a MoS_2_/BP FET with a small positive drain bias. In this case, the current level is already in the 10 to 100 nA range at V_G_ = 0 V and V_D_ = 500 mV, as shown in Figure 2b and Figure 3a. Thus, to turn off this device, a strong negative gate bias should be applied. Many prior studies described band structures similar to Figure 2d to explain the off-state, where the tunneling current does not flow because the carriers in the BP valence band cannot be transferred to the MoS_2_ conduction band. However, as indicated in Figure 2c, the minority carriers from the BP can be injected into the MoS_2_ (and vice versa) when V_G_ is approximately −3 V and the current level is approximately 10 nA. Thus, the reduction of the tunneling component cannot explain the turn off mechanism of our device; i.e., the prior explanation is obviously wrong. 

Thus, the extremely low off current at strong negative gate bias needs to be explained with another mechanism. If we think about the carrier conduction at very negative V_G_, only the electrons from MoS_2_ can be drifted into the BP region and hole drift is blocked by the high barrier as shown in Figure 3b. Then, electrons injected into the BP region recombines holes and form a depletion region. As the V_G_ becomes more negative, the width of depletion region increases further and the drain current decreases rapidly, until the hole diffusion current starts to increase at V_G_ < −4.5 V. Thus, in our opinion, the off-current of the MoS_2_/BP FET can be better explained with the formation of a depletion region in the BP layer. 

If we explain the device operation from the negative V_G_ side, it is easier to understand the operating mechanism. The drain current does not flow at −4.5 V because of the large depletion width. As V_G_ increases to the positive bias side, the depletion region decreases, and suddenly, the minority carriers start drifting to other materials. Then, as the MoS_2_ energy band moves further downward, the tunneling current starts to flow at −3.2 V. Since the tunneling current is added to the drift current, due to the minority carrier injection, the drain current shows a hump at −3.2 V in our device. Most tunnel FETs reported in the literature show this kind of hump in the transfer characteristics, confirming our model. 

**Figure 4:**
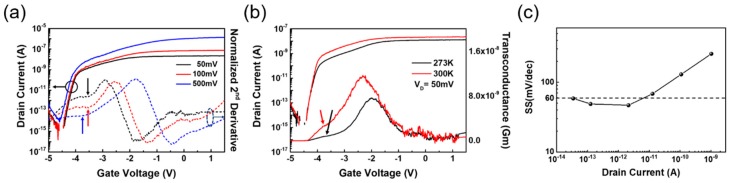
(**a**) Transfer characteristics of a MoS_2_/BP heterojunction FET for different drain voltages (50 mV, 100 mV, and 500 mV). Normalized second derivative of transfer curves are shown to note the initiation points of band-to-band tunneling (BTBT). (**b**) Temperature-dependent transfer characteristic at 273 and 300 K, V_D_ = 50 mV. (**c**) Subthreshold swing (SS) versus drain current at V_D_ = 500 mV, 300 K.

The transfer characteristics measured at different drain biases and temperatures support our operation mechanism model further. When V_D_ is small, the drift current decreases, but the turn-on behavior is not strongly affected because it is more closely related to how the depletion region is formed by the initial band alignment at the BP and MoS_2_ interface. To detect the bias where the BTBT current starts to contribute, the second derivative of the transfer curves is calculated, as shown in Figure 4a. The starting point of the abrupt curvature change marked with an arrow indicates the point of the BTBT current initiation, and the peak position indicates the maximum tunneling current. As the drain bias increases, the band alignment approaches the state shown in Figure 2f. Thus, a higher negative V_G_ should be applied to turn off the tunneling current by pushing the MoS_2_ energy band upward. The temperature dependence also shows an interesting characteristic. The position of tunneling current initiation has not changed significantly, but the peak height increased, indicating that the BTBT current increased due to the increase of thermally activated carriers in the valence band of BP. The drift current increase can be attributed to the increased minority carrier density at the higher temperature.

Finally, we would like to emphasize that our device also shows a sub-60 mV/dec subthreshold swing in some regions of the transfer curve, as shown in Figure 4c. In previous reports (Table 1), swing values below 60 mV/dec often suggest a tunneling mechanism [12,13,16,17,18,19]. However, 60 mV/dec is the limit set by the diffusion mechanism. Since we have proposed that the turn-on behavior of our device is governed by the formation of the depletion region at the MoS_2_/BP interface, the turn-on mechanism is closer to a junction FET, where the drain current starts to flow once a small current path is formed by the reduction of the depletion width. Thus, the swing that is smaller than 60 mV/dec is more closely related to geometric factors and the carrier profile at the BP region, which affect the shape of depletion region.

## 4. Conclusions

In conclusion, we demonstrated the MoS_2_/BP heterojunction FET and analyzed the device operation mechanism. We found that the BTBT is not the primary mechanism determining the on-off characteristics of the MoS_2_/BP heterojunction FET, but it contributes to the formation of the hump in the transfer curve. In addition, the rapid turn-on and extremely low off-current are explained by the depletion region formation. Our results can be applied to general 2D/2D heterojunction devices.

## Figures and Tables

**Table 1:** Comparison of the performance of the 2D/2D tunneling FETs reported in the literature.

Ref.	Material	On Current (A) (V_D_)	I_on_/I_off_ Ratio	SS_MIN_ (mV/dec) at RT	SS_AVG_ (mV/dec) at RT	Dielectric
Our result	MoS_2_/BP	1 × 10^−6^ (500 mV)	~7 × 10^7^	54	94	30-nm Al_2_O_3_ (bottom)
[14]	MoS_2_/p-Ge	5 × 10^−6^ (500 mV)	~8 × 10^7^	3.9	22	Ion gel (top)
[16]	MoS_2_/BP	8 × 10^−6^ (50 mV)	10^6^	55	55	Ion gel (top)
[18]	MoS_2_/WSe_2_	-	-	-	75	10-nm HfO_2_ (bottom)
[19]	MoS_2_/BP	1 × 10^−7^ (800 mV)	~10^4^	-	65	Ion gel (top)
[22]	WSe_2_/SnSe_2_	9 × 10^−7^ (500 mV)	~10^5^	37	80	40-nm Al_2_O_3_ (bottom)
